# Development of Nanobody-Based Sandwich ELISA Resistant to SpA Interference for Sensitive Detection of Staphylococcal Enterotoxin A

**DOI:** 10.3390/bios15100666

**Published:** 2025-10-03

**Authors:** Chenghao Hu, Di Wang, Yangwei Ou, Ruoyu Li, Qi Chen, Peng Liu

**Affiliations:** 1 Department of Clinical Laboratory, The First Affiliated Hospital, Jiangxi Medical College, Nanchang University, Nanchang 330006, China; 2 Second School of Clinical Medicine, Jiangxi Medical College, Nanchang University, Nanchang 330031, China; 3 National Engineering Research Center for Bioengineering Drugs and the Technologies, Jiangxi Provincial Key Laboratory of Bioengineering Drugs, Institute of Translational Medicine, Jiangxi Medical College, Nanchang University, Nanchang 330031, China; 4 Binzhou Center for Disease Control and Prevention, Binzhou 256002, China

**Keywords:** staphylococcal enterotoxin A, nanobody, ELISA

## Abstract

*Staphylococcus aureus* is a major pathogen responsible for staphylococcal food poisoning (SFP), with its pathogenicity primarily dependent on staphylococcal enterotoxins (SEs). Among these, staphylococcal enterotoxin A (SEA) is a critical risk factor due to its high toxicity, high detection rate (accounting for 80% of SFP cases), strong thermal stability, and resistance to hydrolysis. Traditional SEA immunoassays, such as enzyme-linked immunosorbent assay (ELISA), are prone to false-positive results caused by nonspecific binding interference from *S. aureus* surface protein A (SpA). In recent years, nanobodies (single-domain heavy-chain antibodies) have emerged as an ideal alternative to address SpA interference owing to their small molecular weight (15 kDa), high affinity, robust stability, and lack of Fc regions. In this study, based on a previously developed highly specific monoclonal antibody against SEA (mAb-4C6), four anti-SEA nanobodies paired with mAb-4C6 were obtained through two-part (four-round) of biopanning from a naive nanobody phage display library. Among these, SEA-4-20 and SEA-4-31 were selected as optimal candidates and paired with mAb-4C6 to construct double-antibody sandwich ELISAs. The detection limits for SEA were 0.135 ng/mL and 0.137 ng/mL, respectively, with effective elimination of SpA interference. This approach provides a reliable tool for rapid and accurate detection of SEA in food, clinical, and environmental samples.

## 1. Introduction

*Staphylococcus aureus* is resistant to protein hydrolysis, making it difficult to neutralize through conventional food processing methods [1,2,3]. Therefore, developing sensitive and rapid detection methods for SEA is crucial for food safety, clinical diagnosis, and environmental monitoring.

Currently, various methods have been developed for the detection of SEs in different samples, including food, water, and body fluids [4], such as enzyme-linked immunosorbent assay (ELISA) [5], test strips [6], electrochemical analysis [7], and biosensors [8]. Among these, ELISA is the most widely used for rapid SEA screening due to its high throughput, simplicity, and automation. However, staphylococcal protein A (SpA), a surface protein presented in the cell wall of *S. aureus*, can nonspecifically bind to the crystallizable fragment (Fc) region of mammalian (pig, guinea pig, mouse, dog, and monkey, etc.) immunoglobulins, leading to false-positive results in immunoassays detecting SEs using IgG-type antibodies [9,10]. Currently, in order to solve the problem of SpA interference in SEA immunoassays, researchers have replaced conventional IgG with either antigen-binding fragments (Fab) or avian immunoglobulin Y (IgY) [11,12]; however, The Fab is difficult to prepare and the lower affinity of IgY remains a difficult problem to solve.

The nanobodies, also known as single-domain heavy-chain antibodies, are genetically engineered antibodies derived from the variable region of heavy-chain antibodies found in the camelidae family, with a molecular weight of about 15 kDa (one-tenth that of conventional IgG-type antibodies) [13,14,15]. In recent years, nanobodies have gained widespread attention in life science research [16], drug development [17], medical diagnosis [18], food safety [19], environmental monitoring [20], and other fields, mainly due to their small molecular size, high affinity, high stability, good solubility, strong tissue penetration, and recognition of hidden antigenic epitopes [16,21]. Compared to Fab and IgY, nanobodies offer advantages in various properties and may be a promising alternative to address the interference problem of SpA in SEA immunoassays.

**Figure 1:**
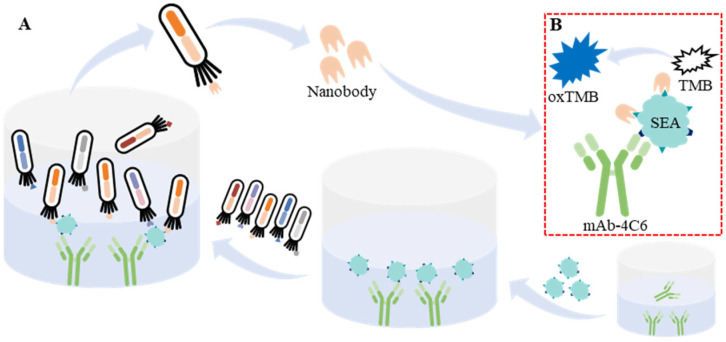
The schematic diagram of biopanning for anti-SEA nanobody paired with mAb-4C6 (**A**) and development of double-antibody sandwich ELISA for the detection of SEA based on mAb and nanobody (**B**).

In the previous work, our laboratory developed a monoclonal antibody (mAb-4C6) with high affinity and specific recognition of SEA, which was used to construct a quantum dot microsphere competitive test strip for the detection of SEA, achieving a detection limit of 1.89 ng/mL for SEA in milk samples [22]. In the present work, in order to avoid the interference of SpA in SEA immunoassay, as illustrated in Figure 1, we obtained six nanobodies paired with mAb-4C6 to bind SEA from a naive nanobody phage display library via two-part (four-round) biopanning and evaluated the various properties (yield, specificity, etc.) of the above nanobodies. Finally, a double-antibody sandwich ELISA was successfully constructed for the detection of SEA resistant to interference by SpA based on the above mAb-4C6 and SEA-4-20 or SEA-4-31; its limit of detection (LOD) for SEA was 0.135 ng/mL and 0.137 ng/mL, respectively. In conclusion, in this work, multiple anti-SEA nanobodies were obtained by screening from a naive nanobody phage display library and used to establish a double-antibody sandwich ELISA against SpA interference, which provides a powerful tool for rapid detection as well as reduction in the harmful effects of SEA.

## 2. Materials and Methods

### 2.1. Materials and Reagents

The phage-displayed naive VHH library (AlpSDAb-P), The Anti-M13 Bacteriophage, AlpHcAbs^®^Rabbit antibody (HRP), The Anti-His tag, AlpHcAbs^®^ Rabbit antibody (HRP), and The Anti-HA tag, AlpSdAbs^®^ VHH (HRP) were obtained from AlpVHHs Co., Ltd. (Chengdu, China). The costar high binding microplate was purchased from Corning Inc. (Corning, NY, USA). The affinity chromatography columns and nickel-nitilotriacetic acid sepharose (Ni-NTA) were purchased from Biogenmicro Biotech (Suzhou, China). The SEA, SEB, SEC, SED, and SEE were purchased from Chiba Biotech (Beijing, China). The TMB two-component substrate solution was purchased from Solarbio (Beijing, China). The pET22b vector, *E. coli* DH5α, and *E. coli* Rosetta were saved in our laboratory. All inorganic chemicals and organic solvents were analytical grade.

### 2.2. The Biopanning of Anti-SEA Nanobodies

The nanobodies against SEA paired with mAb-4C6 were obtained from a phage-displayed naive VHH library (AlpSDAb-P) following a two-stage, four-round biopanning process as described below. In the first part, SEA was used as the target for biopanning. Firstly, SEA (20 μg/mL in 50 mM NaHCO_3_, pH8.6, 100 μL/well) was coated overnight at 4 °C. After washing three times with PBS (10 mM, pH7.4), the microplates were blocked using 3% bovine serum albumin (BSA) or 3% ovalbumin (OVA) at 37 °C for 2 h. After washing three times with PBS (10 mM, pH7.4), the phage-displayed naive VHH library (1 × 10^12^ pfu/mL, 100 μL/well) was preincubated with blocking buffer for 1 h at 37 °C before adding to antigen-coated wells. After incubation at 37 °C for 1.5 h, the unbound phage was removed and washed with PBST and PBS. Then, the bound phages were eluted with 0.1 M Gly-HCl (pH 2.2, 94.5 μL/well) for 8 min and then neutralized with 1 M Tris-HCl (pH 9.0, 5.5 μL/well) immediately. After each round of elution, 10 μL of neutralized phage solution was used for titration to determine phage enrichment, while the remaining neutralized phages (90 μL) were amplified for the subsequent round of biopanning.

To obtain anti-SEA nanobodies paired with the mAb-4C6, two rounds of biopanning were performed using SEA captured by the mAb-4C6 as the target according to the following. Firstly, mAb-4C6 (60 μg/mL in 50 mM NaHCO_3_, pH8.6, 100 μL/well) was coated overnight at 4 °C. After washing three times with PBS (10 mM, pH7.4), the microplates were blocked using 3% bovine serum albumin (BSA) or 3% ovalbumin (OVA) at 37 °C for 2 h. The SEA (10 μg/mL in 10 mM PBS 100 μL/well) was added to mAb-4C6 coated wells to form screening targets. Other steps were performed as above. To screen for obtaining nanobodies against SEA with high affinity paired with mAb-4C6, as shown in Table S1, a progressively more stringent strategy was used to increase elution pressure over the four rounds of biopanning. Afterwards, single colonies were isolated using titration plates of the last two rounds of eluates and identified by phage-ELISA. Phage propagation, titration, and purification were performed according to standard protocols.

### 2.3. Expression and Identification of Anti-SEA Nanobodies

The phagemid vectors containing the anti-SEA nanobody gene were extracted using a plasmid purification kit. and transformed into *E. coli* Rosetta chemically competent cells via heat treatment at 42 °C for 90 s for protein expression according to the following auto-induction method. Then, the transformed cells were cultured on LB agar plates containing ampicillin (100 μg/mL) at 37 °C overnight, and one colony was selected and transferred to 5 mL of LB liquid medium containing 100 μg/mL of ampicillin, and then cultured at 37 °C overnight with 180 rpm shaking. Then, the culture was inoculated into 200 mL of auto-induction medium at 1% inoculum volume and cultured at 37 °C with shaking (180 rpm) until the OD_600_ ≈ 0.6. Subsequently, the auto-induction medium was incubated overnight at 23 °C with shaking at 130 rpm for protein expression. The induced *E. coli* Rosetta cells were centrifuged (6000× *g*, 4 °C, 15 min) and the cell pellet was resuspended in 30 mL of equilibration buffer (20 mM Tric-HCl, 1 M NaCl, 5% Glycerol and 1 mg/mL lysozyme). After enzymatic hydrolysis of the cell walls for 60 min at 4 °C, hydrolyzed *E. coli* Rosetta cells were further disrupted by sonication in an ice bath to avoid degradation or denaturation of the target proteins. The supernatant containing soluble target proteins and a histidine-tag (His-tag) was collected by centrifugation (13,000× *g*, 4 °C, 30 min) and filtered through a 0.22 μm aqueous membrane filter to remove cellular debris. The soluble target protein was then purified using Ni-NTA affinity chromatography according to the manufacturer’s instructions. The eluted fractions were characterized by 12% sodium dodecyl sulfate-polyacrylamide gel electrophoresis (SDS-PAGE) and Western blotting (WB). The concentration of the target proteins was determined by Nanodrop 2000 and stored at −80 °C for further use.

### 2.4. Development of the Sandwich ELISA Based on mAb-4C6 and Nanobody for the Detection of SEA

The proposed sandwich ELISA based on mAb-4C6 and paired nanobodies was performed as follows. Firstly, 100 μL/well of mAb-4C6 was added to the ELISA microplate and incubated at 37 °C for 2 h. After washing three times with PBST (10 mM PBS containing 0.25% Tween 20), 300 μL/well of 1% bovine serum albumin (BSA) was added for block at 37 °C for 2 h. After three washes with PBST, the expressed nanobody proteins were added and incubated at 37 °C for 1 h. After three washes with PBST, 100 µL of HRP-anti-His-tag antibody was added to each well and incubated at 37 °C for 1 h. After three washes with PBST, 100 µL of TMB solution was added to each well and incubated (37 °C, 10 min) to catalyze the TMB substrate. The enzyme-catalyzed reaction was stopped by adding 2 M H_2_SO_4_ (50 μL/well). Finally, the absorbance at 450 nm was measured using a microplate reader.

### 2.5. The Evaluation of SpA Interference Resistance of the Developed Sandwich ELISA

Nanobodies lack the Fc region, which theoretically eliminates interference from SpA when constructing immunoassays for SEA detection. To evaluate the anti-SpA interference effect of the developed sandwich ELISA for SEA detection, we first coated ELISA plates with mAb-4C6 (200 ng/well). After BSA blocking, SEA samples (100 ng/well) with or without the addition of SpA (100 ng/well) were added. Following washing steps, nanobodies were added to form sandwich immune complexes. Finally, detection was performed using HRP-labeled anti-His secondary antibodies.

### 2.6. Practicability of the Developed Sandwich ELISA

Based on the good performance of the proposed sandwich ELISA for the detection of SEA, its practicability was further analyzed in spiked milk samples. Briefly, five concentrations of SEA (5, 15, 45, 135, 405 ng/mL) spiked milk samples were detected by the proposed method and the results were further compared with those of a commercial ELISA kit (JingKang Biological Engineering Co., Ltd. Shanghai, China).

## 3. Results

### 3.1. The Biopanning of Anti-SEA Nanobodies

**Figure 2:**
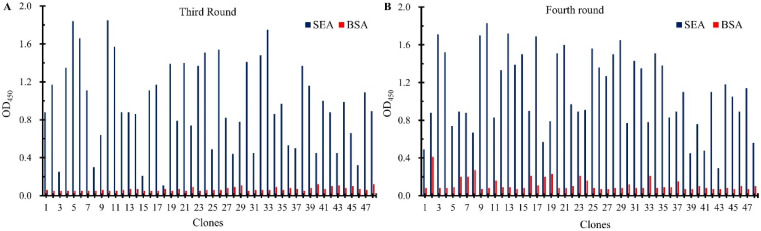
The Identification of anti-SEA nanobodies by the phage-ELISA. In total, 96 clones were selected from the third (**A**) and fourth (**B**) round panning, respectively. An OD_450_ greater than 0.5 (5-fold of BSA) was considered a positive clone and sent for DNA sequencing.

To obtain the nanobodies against SEA paired with mAb-4C6, two-part (four-round) of biopanning were carried out using the SEA or SEA captured by mAb-4C6 as the antigen. The titer of phage in the eluate from each round of elution was tested to assess phage enrichment, as shown in Table 1. Even though the elution conditions became progressively harsher in each round of biopanning, the amount of phage eluted was constantly increasing with enrichments of 132.8, 9.4, and 1.1, which indicated that the nanobodies specifically binding SEA were significantly enriched during the biopanning process. In addition, in order to achieve a diverse and high-affinity of nanobodies, 96 clones were randomly selected from each round (third and fourth) of elution product titer assay plates. The phage-displayed nanobodies were then recovered using M13K07 helper phage rescue, followed by phage-ELISA to assess their binding ability to SEA. As shown in Figure 2, compared to the BSA group, the majority of the 96 tested phage clones exhibited binding activity toward SEA, indicating the successful execution of the phage display library biopanning process. Subsequently, all positive clones were selected for DNA sequencing; as shown in Figure S1, six sequence-specific nanobody sequences were obtained. The great diversity of the above nanobodies was evident by analyzing the amino acid sequences of their three highly variable complementary assay regions (CDR1, CDR2, and CDR3). The relatively conserved FR_2_ framework contains typical hydrophilic amino acid substitutions, which explain the high solubility of VHH as a single structural domain fragment. In addition, the CDR1 and CDR3 regions of nanobodies contain cysteine residues that form disulfide bonds, which contribute to the formation of cyclic structures and play an important role in stabilizing the structure of the antigen-binding region of the nanobodies. The enrichment of the above nanobodies is shown in Table S2, where it can be seen that three strains of nanobodies were enriched multiple times, with SEA-4-03 being the most enriched, presenting a total of 31 times, possibly because it recognizes the major antigenic determinants of SEA or because of its own high multiplication capacity.

### 3.2. The Identification of Anti-SEA Nanobodies

**Figure 3:**
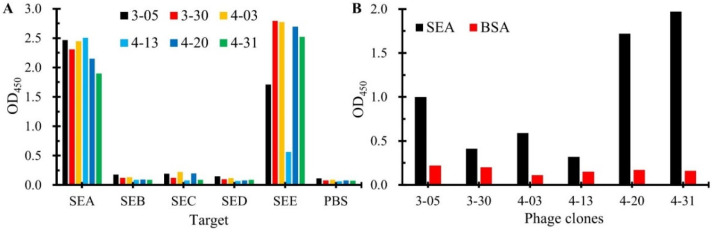
Characterization of seven positive nanobodies. (**A**) Identification of binding ability of six positive nanoantibodies to common SEs. (**B**) Identification of the ability of six positive nanobodies to pair with mAb-4C6 to bind SEA.

The specificity of six nanobodies was tested by phage-ELISA. As shown in Figure 3A, all six nanobodies demonstrated binding to SEA. Notably, five exhibited strong cross-reactivity with SEE, while SEA-4-13 showed relatively weaker binding to SEE. Importantly, none of the nanobodies bound to other tested SEs (SEB, SEC, SED). Sequence alignment analysis of SEs (Figure S2) revealed that SEE shares the highest amino acid sequence similarity (>90%) with SEA, which may explain the observed cross-reactivity with SEE. This finding suggests the necessity of incorporating negative screening against other SEs during subsequent development of SEA-specific nanobodies to eliminate those targeting conserved epitopes among SEs.

To identify anti-SEA nanobodies compatible with mAb-4C6 for sandwich assays, we performed phage-ELISA pairing tests. Briefly, the ELISA plates were coated with mAb 4C6 (2 μg/mL, 100 μL/well) followed by BSA blocking. After adding SEA antigen (0.5 μg/mL, 100 μL/well) and washing, phage-displayed nanobodies were applied sequentially with HRP-conjugated anti-M13 secondary antibody. As shown in Figure 3B, three nanobodies (SEA-3-05, SEA-4-20, SEA-4-31) demonstrated significantly higher OD_450_ values in the SEA group compared to BSA controls, indicating their ability to bind the SEA antigen captured by the mAb-4C6. This result confirms that these three nanobodies exhibit ideal pairing compatibility with the mAb-4C6 in the SEA detection system. In contrast, the other three nanobodies (SEA-3-30, SEA-4-03, SEA-4-13) showed weak signal enhancement, suggesting that steric hindrance effects may exist between mAb-4C6 and these nanobodies when antigens are simultaneously bound, making it difficult to form sandwich immunocomplex.

### 3.3. Expression and Characterization of Anti-SEA Nanobodies

**Figure 4:**
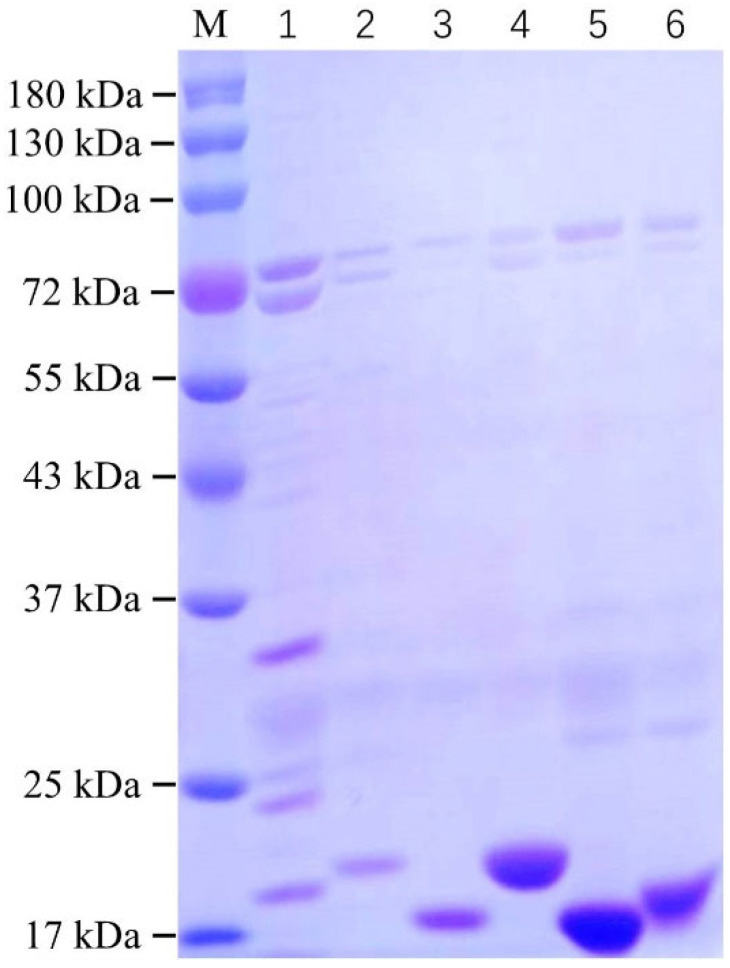
SDS-PAGE identified six nanobodies protein. Line M: prestained protein marker (11–180 kDa); Line 1: SEA-3-05 nanobody; Line 2: SEA-3-30 nanobody; Line 3: SEA-4-03 nanobody; Line 4: SEA-4-13 nanobody; Line 5: SEA-4-20 nanobody; Line 6: SEA-4-31 nanobody.

The recombinant pcomb3xss plasmid containing the anti-SEA nanobody gene was transformed into Rosetta *E. coli* competent cells. Subsequently, six high-purity anti-SEA nanobody proteins were obtained through auto-induction expression, nickel column purification, and dialysis. The purified nanobodies were characterized using SDS-PAGE, as shown in Figure 4. The molecular weights of all six purified anti-SEA nanobodies were approximately 17 kDa. Notably, the nanobody SEA-3-05 exhibited higher levels of other proteins, and its purity showed no significant improvement even after optimization of the purification conditions. Due to challenges in preparation, the nanobody SEA-3-05 was excluded from further testing in subsequent experiments. In contrast, the remaining five nanobodies demonstrated purities exceeding 80%. Among these, SEA-4-13, SEA-4-20, and SEA-4-31 displayed particularly outstanding performance in both concentration and purity. We further characterized the purified nanobodies using Western blot (WB) analysis; as shown in Figure S3, all purified nanobodies exhibited molecular weights close to their theoretical values, indicating that our purification strategy for these nanobodies was effective. The expression levels of all six nanobodies are summarized in Table S3.

### 3.4. The Quantitative Detection Curves of the Development Sandwich ELISA

**Figure 5:**
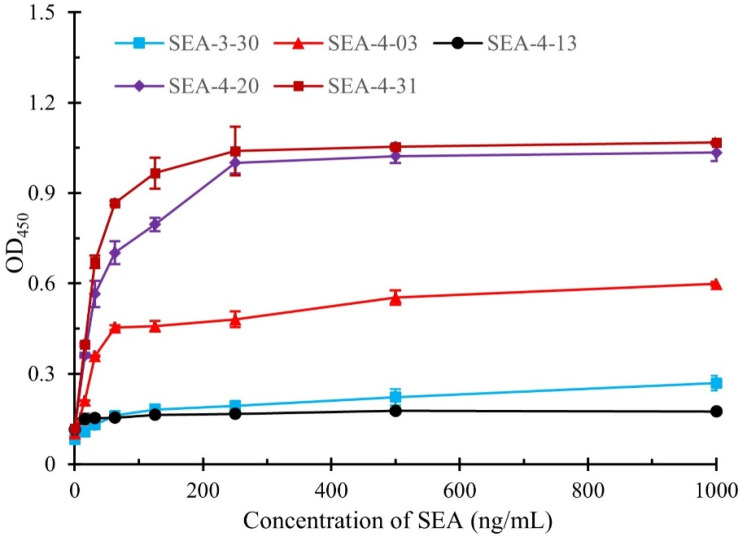
The detection curves of the developed sandwich ELISA for quantitative detection of SEA. The error bars represent the standard deviation of triplicate sample tests.

To evaluate the capability of the obtained nanobodies to pair with mAb (4C6) in the development of sandwich immunoassay, the SEA samples at varying concentrations were tested and the quantitative detection curves were constructed. As shown in Figure 5, the nanobodies SEA-4-20 and SEA-4-31 demonstrated superior detection performance compared to other nanobodies, with limits of detection (LOD) of 0.135 ng/mL and 0.137 ng/mL, respectively. In contrast, the SEA-4-03 nanobody exhibited poor detection efficacy, likely attributable to its lower binding affinity relative to the aforementioned two nanobodies. Additionally, the nanobodies SEA-3-30 and SEA-4-13 displayed low absorbance even at elevated SEA concentrations, which may stem from steric hindrance caused by mAb-SEA binding interfering with nanobody recognition, consistent with previous findings. The linear relationship between the SEA concentration ranging from 0.9765 to 62.5 ng/mL and the OD_450_ values is illustrated in Figure S4. This relationship exhibits a reliable correlation coefficient. Table S4 exhibits a comparative of the developed sandwich ELISA with previously reported assays for SEA detection. It can be seen, in comparison with previous biosensors for determining SEA, that the presented sandwich ELISA provides high sensitivity. Based on these results, the nanobodies SEA-4-20 and SEA-4-31 were selected for subsequent experiment.

### 3.5. Analysis of SpA Interference Resistance of the Developed Sandwich ELISA

Anti-SpA interference constitutes a critical advantage of the nanobody-based immunoassay for SEA detection. To assess the interference resistance of the developed immunoassay against SpA, the SEA samples spiked with SpA were analyzed. As shown in Figure S5A, the absorbance values of SpA-spiked SEA samples showed a slight reduction compared to the control group, indicating minor interference from SpA. This phenomenon may be attributed to the utilization of mAbs (4C6) as capture antibodies in the developed sandwich ELISA. To completely eliminate SpA interference, we plan to conduct pairwise analysis of six nanobodies and develop a nanobody-based sandwich immunoassay for the detection of SEA.

The specificity of the developed sandwich ELISA was validated against common SEs (SEA, SEB, SEC, SED, SEE). As shown in Figure S5B, they demonstrate exclusive recognition of SEA with no cross-reactivity to other SEs, consistent with the high specificity of the mAb-4C6 employed. This finding aligns with our previous studies and confirms the excellent specificity of the developed sandwich ELISA for the detection of SEA.

### 3.6. Practicability of the Developed Sandwich ELISA

**Figure 6:**
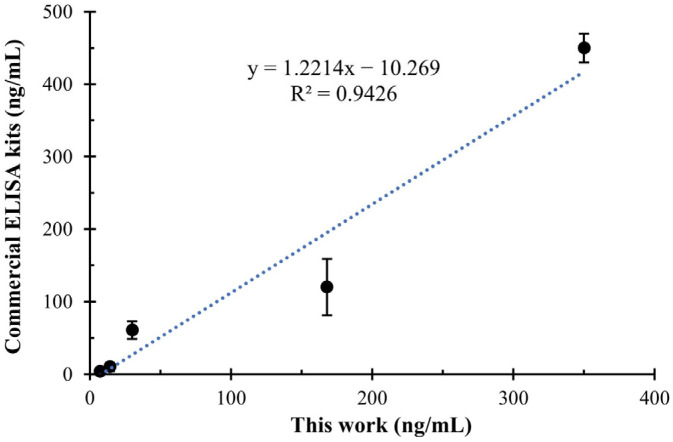
The correlation analysis between the proposed sandwich ELISA and commercial ELISA kits for the quantitative detection of SEA in real samples.

To evaluate the practicality of the developed sandwich ELISA, we applied it to detect SEA-spiked milk samples. We found that diluting milk samples by more than 5-fold effectively reduced matrix effects on results. Considering the linear range of the proposed detection method, a series of SEA standard (5, 15, 45, 135, 405 ng/mL) were added to milk samples for testing. The results were further compared with commercial ELISA kits. As shown in Figure 6, the developed sandwich ELISA demonstrated excellent consistency (R^2^ = 0.9426) with the commercial ELISA kits, confirming the practicality of the developed sandwich ELISA in real samples.

## 4. Conclusions

This work successfully isolated six SEA-binding nanobodies through a two-part (four-round) of biopanning from a naive nanobody phage display library. Characterization revealed that over 80% of these nanobodies could pair with mAb-4C6 for cooperative SEA recognition, demonstrating that our stepwise panning strategy significantly improves the success rate of identifying complementary nanobody pairs. Key characteristics including production yield and specificity were systematically evaluated for all six nanobodies. A sandwich ELISA detection platform was subsequently established using mAb-4C6 in combination with two optimized nanobodies (SEA-4-20 and SEA-4-31), achieving a LOD of 0.135 ng/mL and 0.137 ng/mL, respectively. Notably, the nanobody-based sandwich ELISA effectively circumvented the false-positive interference caused by nonspecific binding of staphylococcus SpA to traditional IgG antibodies.

In summary, this work achieved two major advancements: (1) identification of six novel SEA-specific nanobodies through phage display library screening, and (2) development of a nanobody/mAb-based sandwich ELISA method for SEA detection. This methodology not only enhances food safety monitoring capabilities against SEA contamination, but also provides a new paradigm for detecting biological toxins in clinical diagnostics and environmental surveillance. Future investigations should focus on optimizing nanobody pairing configurations for SEA recognition, improving binding affinity through affinity maturation or multivalent formats, and exploring integration with emerging biosensing technologies to facilitate development of portable detection devices.

## Figures and Tables

**Table 1:** The process monitoring of the anti-SEA nanobodies panning.

Round	Library Input (pfu/well)	Library Output (pfu/well)	Recovery Rate	Enrichment Ratio
1	1 × 10^11^	6.4 × 10^4^	6.4 × 10^−7^	/
2	1 × 10^11^	8.5 × 10^6^	8.5 × 10^−5^	132.8
3	1 × 10^11^	8.0 × 10^7^	8.0 × 10^−4^	9.4
4	1 × 10^11^	8.4 × 10^7^	8.4 × 10^−4^	1.1

Recovery rate = Library Output/Library input; Enrichment Ratio = Next round recovery/Last round recovery.

## Data Availability

Data is contained within the article or Supplementary Materials.

## Supplementary Materials

The following supporting information can be downloaded at https://www.mdpi.com/article/10.3390/bios15100666/s1; Figure S1: The amino acid sequence of six positive nanobodies; Figure S2: The amino acid sequence of SEs; Figure S3: The western blot identified positive nanobodies protein; Figure S4: The detection curves of the developed sandwich ELISA for quantitative detection of SEA. The error bars represent the standard deviation of triplicate sample tests; Figure S5: The performance analysis of the developed sandwich ELISA. A: Anti-SPA interference analysis; B: Specificity analysis; Table S1: The biopanning conditions for anti-SEA nanobodies; Table S2: The occurrence frequency of six anti-SEA nanobody clones in the third and fourth rounds of biopanning; Table S3: The expression yield of six nanobodies; Table S4: Comparison of the developed sandwich ELISA with previously reported assays for SEA detection. References [23,24,25,26,27,28,29] are cited in Supplementary Materials.
