# Supplementary material for: Development of Nanobody-Based Sandwich ELISA Resistant to SpA Interference for Sensitive Detection of Staphylococcal Enterotoxin A
Source: Biosensors (Basel). 2025 Oct 3;15(10):666. doi: 10.3390/bios15100666 (PMC12564327; doi:10.3390/bios15100666)
Supplement: Supplementary file 1 [file biosensors-15-00666-s001.zip › biosensors-3809376-supplementary.pdf]

Supporting Information

Development of Nanobody-Based Sandwich ELISA Resistant to SpA Interference for Sensitive Detection of Staphylococcal Enterotoxin A

Chenghao Hu[a,b,c,#], Di Wang[c,d,#], Yangwei Ou[c], Ruoyu Li[c], Qi Chen[c], Peng Liu[a,*]

[a] Department of Clinical Laboratory, The First Affiliated Hospital, Jiangxi Medical College, Nanchang University, Nanchang 330006, China;

[b] Second School of Clinical Medicine, Jiangxi Medical College, Nanchang University, Nanchang 330031, China;

[c] National Engineering Research Center for Bioengineering Drugs and the Technologies, Jiangxi Provincial Key Laboratory of Bioengineering Drugs, Institute of Translational Medicine, Jiangxi Medical College, Nanchang University, Nanchang 330031, China;

[d] Binzhou Center For Disease Control And Prevention, Binzhou, 256002, China.

[#] These authors contributed equally to this work.

Table of contents

Table S1 The biopanning conditions for anti-SEA nanobodies……………………………………..S-3

Table S2 The occurrence frequency of six anti-SEA nanobody clones in the third and fourth rounds of biopanning ………………………………………………………………………………………..S-4

Table S3 The expression yield of six nanobodies…………………………………………………S-5

Table S4 Comparison of the developed sandwich ELISA with previously reported assays…………S-6

Figure S1 The amino acid sequence of six positive nanobodies………….………………….………S-7

Figure S2 The amino acid sequence of SEs……………….…………………………….…………….…..S-8

Figure S3 Western blot identified positive nanobodies protein…………………………………...S-9

Figure S4 The detection curves of the developed sandwich ELISA for quantitative detection of SEA…………………………………………………………………………………………....S-10

Figure S5 The performance analysis of the developed sandwich ELISA…………………………....S-11

Table S1. The biopanning conditions for anti-SEA nanobodies

Round	Antibody (μg/well)	SEA (μg/well)	Blocking Buffer	Incubation Time (h)	Library Input (pfu/well)	Tween-20 Concentration	Washing Times
1	/	2.0	3%BSA	1.5	1×10^{11}	0.25%	5
2	/	1.5	3%OVA	1.0	1×10^{11}	0.50%	10
3	6	1.0	3%BSA	1.5	1×10^{11}	0.25%	5
4	6	0.5	3%OVA	1.0	1×10^{11}	0.75%	15

Table S2. The occurrence frequency of six anti-SEA nanobody clones in the third and fourth rounds of biopanning

Number	3-5	3-30	4-3	4-13	4-20	4-31
Third Round	4	2	11	0	0	0
Fourth round	1	0	20	1	1	1
Total	5	2	31	1	1	1

Table S3. The expression yield of six nanobodies

Number	3-5	3-30	4-3	4-13	4-20	4-31
Yield (mg/L)	1.7	1.5	2.1	4.5	4.8	3.6

Table S4 Comparison of the developed sandwich ELISA with previously reported assays for SEA detection

Signal	Recognition elements	Samples	LOD	References
Colorimetry	mAb	Milk	0.061 ng/mL	[1]
Electrochemical	Aptamer	Milk, Meat, Serum	0.34 ng/mL	[2]
Colorimetry	Nanobodies	Milk, Pork	0.43 ng/mL	[3]
Colorimetry, Fluorescence	Nanobodies	Milk, Pork	0.09～0.4 ng/mL	[4]
Fluorescence	Aptamer	Milk	0.899 ng/mL	[5]
Light scattering	mAb	Meat, Vegetables, Milk	10.39 pg/mL	[6]
LSPR	mAb	Milk	5 ng/mL	[7]
QCM	Molecularly imprinted polymer	Milk	2.25 ng/mL	[8]
Colorimetry	mAb, Nanobody	Milk	0.135-0.137 ng/mL	This work

References

1. Duan, H.; Zhao, L.; Wang, J.; Wang, X.; Zheng, L.; Huang, X. Integrating lateral flow device with controllable gold in situ growth for sensitive detection of staphylococcal enterotoxin A in milk. *Analytica Chimica Acta* **2024**, *1329*, 343233.
2. Nodoushan, S.M.; Nasirizadeh, N.; Sedighian, H.; Kachuei, R.; Azimzadeh-Taft, M.; Fooladi, A.A.I. Detection of Staphylococcal Enterotoxin A (SEA) using a sensitive nanomaterial-based electrochemical aptasensor. *Diamond and Related Materials* **2022**, *127*, 109042.
3. Cui, Y.; Wang, X.; Wu, H.; Zhang, X.; Xu, Y.; Yu, G.; Liu, X.; Yao, Q.; Wang, J.; Ji, Y. A" one to two" novel sandwich immunoassay based on nanobodies for detection of staphylococcal enterotoxin A in food samples. *Food Control* **2024**, *160*, 110313.
4. Zhang, Y.; Liu, D.; Tian, Y.; Li, M.; Li, Y.; Zhou, T.; Zhao, Q.; Zhang, M.; Yu, Y.; Pan, H. Bifunctional nanobody facilitates a colorimetric and fluorescent dual-mode immunoassay of Staphylococcal enterotoxin A. *Food Chemistry* **2025**, *467*, 142362.
5. Ma, X.; Meng, R.; Yu, M.; Guo, N.; Wang, H.; Zheng, H.; Sun, C. Label-free and low-background fluorescent structure-switching aptasensor for sensitive detection of staphylococcal enterotoxin A based on graphene oxide-assisted separation of ssDNA. *Food Control* **2024**, *155*, 110105.
6. Tong, W.; Du, Y.; Yao, M.; Fang, H.; He, W.; Zhang, Y.; Su, Y.; Leng, Y.; Huang, X.; Xiong, Y. Gold nanocubes etching enhanced light scattering immunoassay for highly sensitive detection of Staphylococcus aureus enterotoxin A. *Food Chemistry* **2025**, *479*, 143713.
7. Ben Haddada, M.; Hu, D.; Salmain, M.; Zhang, L.; Peng, C.; Wang, Y.; Liedberg, B.; Boujday, S. Gold nanoparticle-based localized surface plasmon immunosensor for staphylococcal enterotoxin A (SEA) detection. *Analytical and bioanalytical chemistry* **2017**, *409*, 6227-6234.
8. Liu, N.; Li, X.; Ma, X.; Ou, G.; Gao, Z. Rapid and multiple detections of staphylococcal enterotoxins by two-dimensional molecularly imprinted film-coated QCM sensor. *Sensors and Actuators B: Chemical* **2014**, *191*, 326-331.

```
      Q V Q L V E S G G G L V Q P G G S L R L S C A A S - - G X L L D H I A I G W F R
      1                 10                  20                  30                  40
3-05  · · · · · · · · · · · · · · · · · · · · · · · A P - - G R I · A A · G · A · · · ·
3-13  E V · · · · · · · · · · E · · · · · · · · · · A A - - G F I · · · · · · A · · · ·
4-03  · · · · · · · · · · · · · · · · · · · · · · · A S - - G S S · Q S · · · · · · · ·
4-13  E V · · · · · · · · · · · · · · · · · · · · · H A D S G R T · · A · G · · · · ·
4-20  · · · · · · · · · · A · · · · · · · · · · · V A S - - G S V F · · · · · · · · ·
4-31  · V · · · · · · · · · · · · · · · · · · · · T D S A R G V S · · · · · · · · · ·

      Q X P G K E R E G V S C I S S R G G D F T N Y A D S V K G R F T I S R D N A K N
                        50                  60                  70                  80
3-05  · V · · · · · · A · G · · · N S G P D F · · · · · · A · · · · · · · · · I S · H
3-13  · V · · · · · · · · · · M · R S G - - V · · · · · · · · · · · · · · · · D · E K
4-03  · A · · · · · D R · · · · · S T T D E T · Y · · · · · · · · · · V · · · · V · ·
4-13  · V · · · · · · · I · · · · N R G G D F A · · F G D A · · · · · V G · · T S T ·
4-20  · A · · · · · · · · · S · · - R S G G I · K · S · · · · · · · · · · · · · · · ·
4-31  · A · · · · · · A · · · · · - R S G R N · A I · · · · · · · · · · · · · · · M ·

      T V Y L Q M N S L K P E D T A V Y Y C A A D X T P P W T L X X X X E S Q Y D Y W
                        90                  100                 110                 120
3-05  · · · · · · · · · · · · · · · I · · · · · D Y C P P W T L T S I P A S K · · S ·
3-13  · L · · · · A · · · D · · · · · · · · · · E A S P S S S L C W V T E - Q · G · ·
4-03  · · S · · · · · · · D · · · · · · · · · T V G T Y C G G S H Y Y - P H Q · · · ·
4-13  · · · · · · · D · · · · · S G I · F · · T D Y C P P W T L S G I H E S R · · T ·
4-20  · · · · · · · · · · · · · · · L · · · · · A A T P W E T P I I S P M S E · G · ·
4-31  · · T · · · · · · N · · · · · · · I · · · R P T P F N E C E M S - E G W F T · ·

      G G G T Q V T V S S X X X X X X P X -
                        130                 140
3-05  · · · · · · · · · A A H H S E D · S *
3-13  · · · · · · · · · S A H H S E D · S *
4-03  · · · · · · · · · L E P K T P K · Q P *
4-13  · · · · · · · · · S A H H S E D · S *
4-20  · · · · · · · · · S E P K T P K · Q P *
4-31  · · · · · · · I · S E P K T P K · Q D *
```

Figure S1. The amino acid sequence of six positive nanobodies

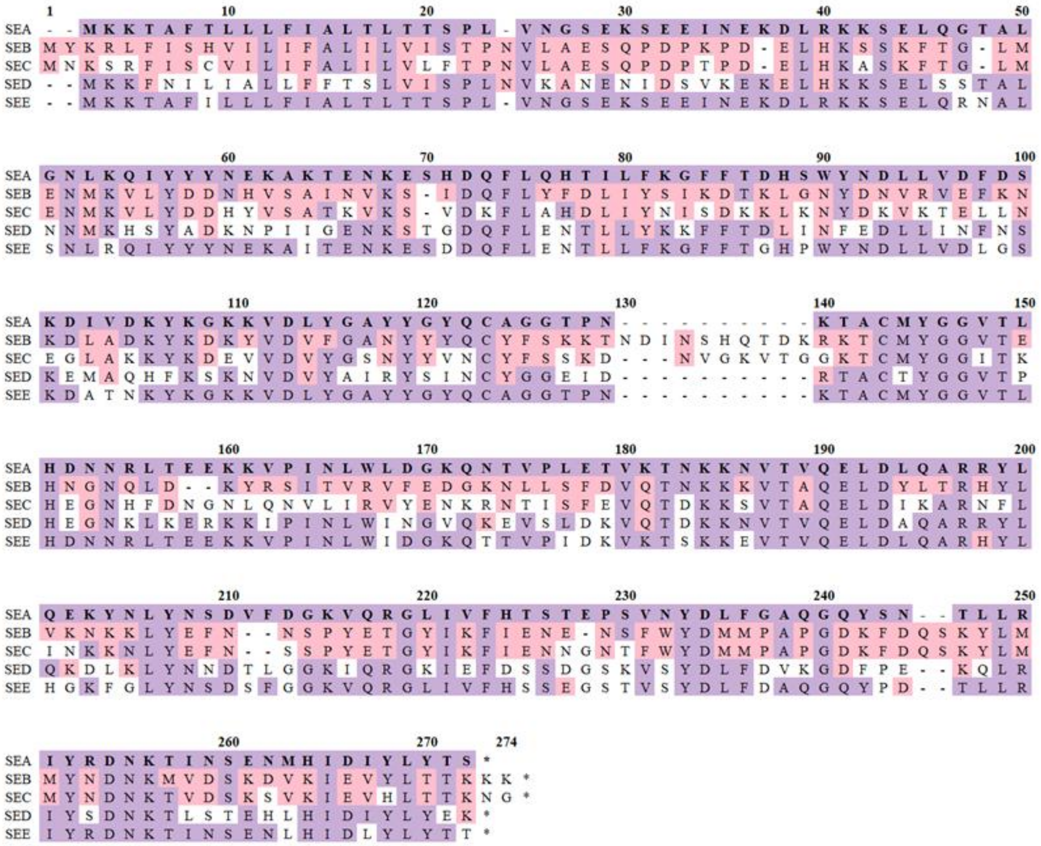

Figure S2. The amino acid sequence of SEs

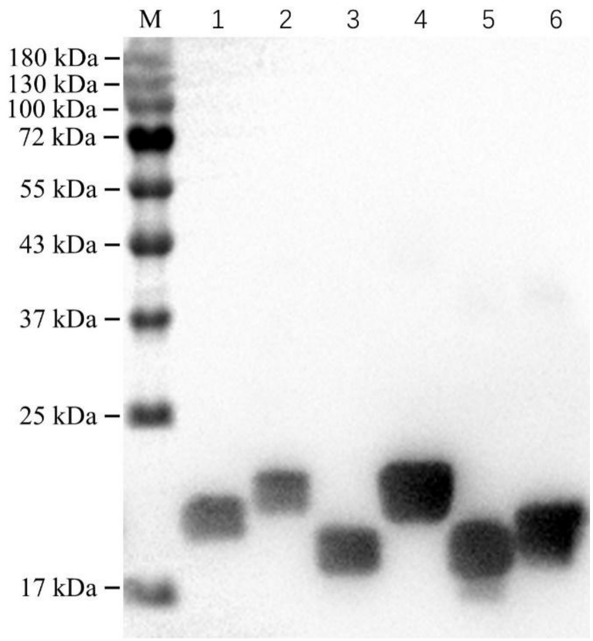

Figure S3. The western blot identified positive nanobodies protein

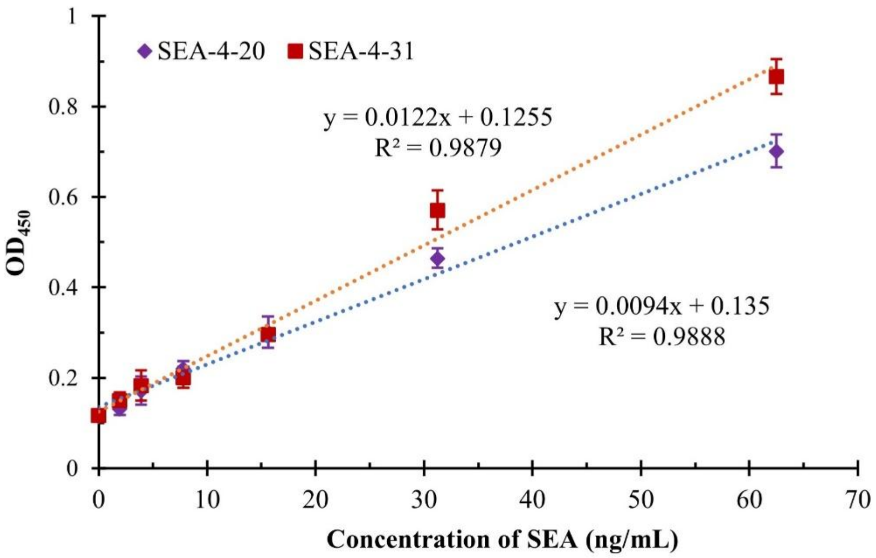

Figure S4. The detection curves of the developed sandwich ELISA for quantitative detection of SEA. The error bars represent the standard deviation of triplicate sample tests.

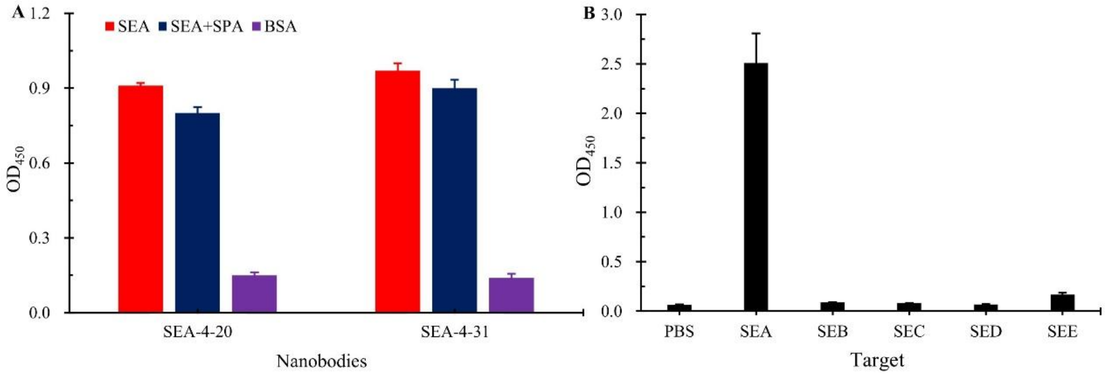

Figure S5. The performance analysis of the developed sandwich ELISA. A: Anti-SPA interference analysis; B: Specificity analysis.
